# Overcoming barriers to drug development and enrollment in clinical trials for adolescents and young adults with lymphoma

**DOI:** 10.1002/jha2.787

**Published:** 2023-09-07

**Authors:** Keri Toner, Carl E. Allen, Shweta Jain, Brad Kahl, John Leonard, Heather Wasserstrom, Jonathan W. Friedberg, Nita L. Seibel, Kara Kelly

**Affiliations:** ^1^ Center for Cancer and Blood Disorders Children's National Hospital Washington District of Columbia USA; ^2^ Baylor College of Medicine Texas Children's Hospital Houston Texas USA; ^3^ Clinical Development Seagen Inc. Bothell Washington USA; ^4^ Department of Medicine Division of Oncology Washington University in St. Louis School of Medicine St. Louis Missouri USA; ^5^ Weill Department of Medicine Division of Hematology and Medical Oncology Weill Cornell Medicine New York New York USA; ^6^ Clinical Development Team Pediatrics Hematology and Cellular Therapy at Bristol Myers Squibb New York New York USA; ^7^ Wilmot Cancer Institute University of Rochester Rochester New York USA; ^8^ Division of Cancer Treatment and Diagnosis National Cancer Institute National Institutes of Health Bethesda Maryland USA; ^9^ Roswell Park Comprehensive Cancer Center University at Buffalo Jacobs School of Medicine and Biomedical Sciences Buffalo New York USA

**Keywords:** adolescent and young adults, clinical trials, lymphomas, new drug development

## Abstract

Lymphoma is one of the most common cancers in adolescents and young adults, but historically, this population has had lower clinical trial enrollment and improvements in overall survival as compared to other age populations. There are multiple challenges that are unique to this population that have affected drug development and clinical trial enrollment. Our panel of experts have identified barriers, and in this review, we discuss current methods to address these barriers as well as potential solutions moving forward.

## Graphical abstract

There are multiple unique challenges to drug development and clinical trial enrollment for adolescent and young adult (AYA) patients with lymphoma. Our panel of experts including consortia leaders, AYA advocates, and industry representatives have identified at least six main issues that have contributed to the inadequacy of novel agents and clinical trials specifically targeting or including the AYA population. Some identified solutions include collaboration between stakeholders and among networks as well as support for providers and patients to improve enrollment. Ultimately overcoming these barriers would allow increased knowledge of the biology of lymphoma subtypes within the AYA population and improve outcomes for this vulnerable population.

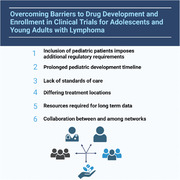

## 1 INTRODUCTION

Lymphoma is one of the most common cancer diagnoses in adolescents and young adults (AYA), accounting for about 20%–25% of cancer diagnoses in this age range (defined as age 15–39 years). Despite overall improvements in survival, the AYA population has not historically experienced as robust survival gains as compared to other age groups [1, 2, 3, 4]. There are multiple unique challenges for the AYA population with lymphoma: compared to other age groups, the AYA population has disparities in access to health insurance, unique psychosocial challenges, differences in disease biology, and importantly, historically low rates of enrollment in clinical trials [5, 6]. Low rates of enrollment are partially due to a lack of accessibility of clinical trials targeted specifically for the AYA population, as well as various barriers to enrollment on trials that are available, although significant strides have been made to improve trial access and accrual at the national level [7]. In this review, we will discuss challenges to drug development for AYA patients with lymphoma, disparities in clinical trial enrollment, and methods to overcome these challenges.

There is a significant need for participation of AYAs in clinical trials in order to increase knowledge of differing lymphoma disease biology, evaluate novel agents, identify age‐specific toxicities and long‐term effects, and improve overall outcomes for this patient population [1]. Therefore, specifically targeting and including AYAs with lymphoma is imperative to multiple stakeholders, including regulatory agencies, sponsors, academic institutions, community providers, patients, and patient advocates. There are a variety of methods by which stakeholders can increase access, inclusion, and enrollment for AYA patients. The infrastructure of research and drug development from the initiation of concept development through trial completion should include thoughtful methods to include and target the AYA population.

## 2 BARRIERS TO AYA DRUG DEVELOPMENT AND ENROLLMENT IN CLINICAL TRIALS

By better understanding the barriers and potential solutions, stakeholders can collaborate to improve enrollment and eventually outcomes. Because this population spans a range between childhood and adulthood, inherently, these patients are treated at both pediatric and adult institutions with differing regulatory complications and treatment methods, which complicates matters and requires additional resources. Our panel of experts, including consortia leaders, AYA advocates, and industry representatives, have identified at least six main issues that have contributed to the inadequacy of novel agents and clinical trials specifically targeting or including the adolescent and young adult population. The following are identified barriers to address.

### 2.1 Inclusion of pediatric patients imposes additional regulatory requirements

Inclusion of pediatric patients involves additional regulatory requirements above what is required for trials that include patients only over age 18 years, potentially prohibiting access for some of the younger AYA population to early clinical trials. In addition, regulatory agencies have historically required safety data on investigational agents in adults before permitting pediatric participation (age <18 years) in clinical trials. Ideally, younger adolescents would be included upfront in clinical trials of novel agents, rather than waiting for adult data to be generated before pursuing adolescent and pediatric development. This requires a concerted effort by both adult and pediatric experts to think more inclusively when designing clinical studies and entails more robust communication between AYA‐focused clinicians, institutional review boards (IRBs), and regulatory agencies on the ethical inclusion of adolescents upfront. The automatic exclusion of patients under 18 years diminishes the ability to further research AYA‐specific disease biology or outcomes in addition to being a disservice to individual patients. If adolescent patients cannot be included upfront, a timeline should be included in planning of a trial of when they can be included.

The National Cancer Institute—Children's Oncology Group Pediatric Molecular Analysis for Therapy Choice (NCI‐COG Pediatric MATCH) trial provided a national framework for trials of molecularly targeted therapies for patients aged 1–21 years with identified targetable alterations [8]. AYAs made up 40.6% of the initial 1000 patients enrolled, but lymphomas/histiocytosis accounted for only about 3% of the diagnoses. The screening protocol allowed for patients to successfully match and enroll in clinical trials with investigational agents. The trial was broadly accessible and allowed for access for both pediatric and young adults to early clinical trials [8]. The pediatric MATCH trial is an example of a creative umbrella approach to successfully improve access to early clinical trials for patients, especially AYA patients with rare tumors [9].

### 2.2 Prolonged pediatric development timeline

In addition to the increased regulatory requirements described as delaying pediatric access to investigational agents, the AYA population also has the potential for slower accrual in clinical trials. There are times that blanket requirements force less than ideal studies to be designed and slow down approvals instead of accelerating them. The historically slow enrollment of AYA patients and can have negative consequences by prolonging study completion, extending the overall drug development program and ultimately delaying approval and access to treatment for any population. Whether pediatric and AYA patients should be included in adult development, a parallel development path, a staggered development path or not at all is best determined on a case‐by‐case basis in consultation between sponsors, regulators, and advising experts given the agent, the indication(s) being considered, the degree to which unmet needs are being met, and the operational structure that will allow for the most accelerated development plan.

Efforts should include enrolling enough adolescents and young adults in studies so that they are sufficiently represented to permit robust data analysis. Collaboration and partnership between pediatric and adult institutions is needed, as well as cooperation of study sponsors and the recommendation of regulatory agencies. Therefore, novel methods for recruiting, retaining, and supporting these patients are needed, in conjunction with mechanisms to allow for continued, unhindered development in other populations, for the best interest of accelerating drug development for all patients.

### 2.3 Lack of standards of care

Treatment practices differ between pediatrics and adults for a variety of lymphoma subtypes, which would need to be reconciled to run a joint trial. Subgroups of non‐Hodgkin lymphoma (NHL) and Hodgkin lymphoma (HL) include an array of rare lymphoma subtypes that further complicate matters. While basket study designs are helpful, it is important that B‐ and T‐cell malignancies are not lumped together and that sufficient numbers of AYAs are enrolled such that distinct analyses can be made within AYA and adult lymphoma subtypes.

An established standard of care and clear criteria for response will build confidence for sponsor‐led studies for lymphoma disease subtypes. It will be important for researchers and clinicians (including both pediatric and adult clinicians) to develop these approaches in order to more effectively work with sponsors and regulators regarding trial specifics [4]. Where differences in standards of care exist among adult groups, or between pediatrics and adults, clinical trials that allow for a limited number of therapeutic backbones with a common experimental question may increase opportunities for collaboration. For example, the current primary mediastinal B‐cell lymphoma study open through the Children's Oncology Group and adult National Clinical Trials Network (NCTN) groups randomizes nivolumab, but allows for three potential treatment backbones based on institution/physician preference (NCT04759586).

### 2.4 Differing treatment locations

Clinics treating pediatric and adult patients differ, requiring increased sites and resources to support a trial involving the AYA population. Also, many AYA patients are treated at local community sites without access to sponsor‐led or investigator‐led trials at academic institutions [10]. Even within institutions, enrollment in clinical trials may depend on the providers themselves. Prior studies have demonstrated that patients treated by a pediatric provider were over 10 times more likely to be enrolled in a clinical trial than an adult provider within an academic institution [11].

While the AYA population in general needs increased enrollment and accessibility to clinical trials, enhanced efforts to reduce disparities in patients with lymphoma based on race and/or ethnicity within the AYA demographic are also warranted [2, 4]. A Surveillance, Epidemiology and End Results (SEER)‐based analysis of outcomes by race and ethnicity in children and AYAs with HL demonstrated that black AYAs had worse survival than white AYAs (white 91.1% [95% confidence interval, CI 89.7–92.3] compared to black 80.5% [95% CI 75.5–84.6]) [4, 12]. A contributing factor may be decreased clinical trial enrollment. Utilizing NCI's cancer therapy evaluation program and SEER data, the enrollment of black patients was evaluated, identifying that between 2000 and 2015, <3% of black AYAs diagnosed with cancer enrolled in cancer treatment trials. The lowest enrollment was for black AYA males [13].

The NCI Community Oncology Research Program (NCORP) was created to improve cancer clinical trial (CCT) enrollment within community‐based oncology and minority‐serving sites in the United States and includes 46 sites [14]. Yet despite this program, identified barriers at NCORP sites include insufficient staff and resources, trial availability and eligibility, physician gatekeeping, uncertainty regarding enrollment process, communication between pediatric and adult oncology, and the finances and regulatory burden [5].

### 2.5 Resources required for long‐term data

Expectations for long‐term follow‐up (LTFU) data for pediatric and adolescent patients are resource intensive and require assistance and persistence from every facet of our oncology community to pursue over the years of interest of a clinical trial. Since LTFU and survivorship care go beyond the duration of any one trial and is a concern for all stakeholders, community‐wide creative solutions are required to collect meaningful data.

Data collection in new studies should be planned with the AYA population in mind so that data can be aggregated and analyzed to provide as much information about this population from the trial. The data collection and analysis should include long‐term effects in addition to acute toxicities, with the inclusion of evaluations for disparities between and among populations [15, 16].

### 2.6 Collaboration between and among networks

**FIGURE 1:**
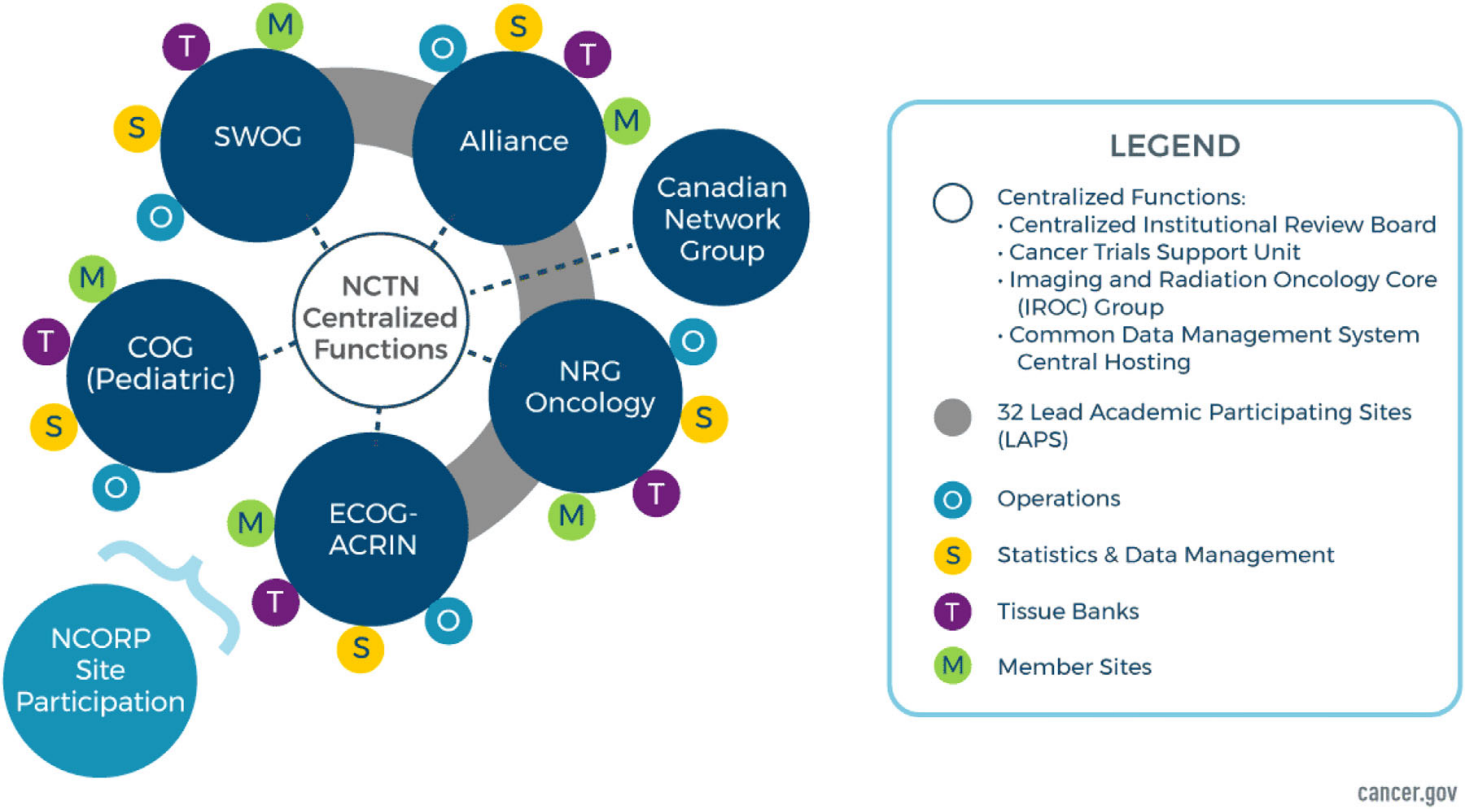
National Cancer Institute (NCI) National Clinical Trials Network (NCTN) structure. Taken with permission from NCI website https://visualsonline.cancer.gov/searchaction.cfm?q=nctn.

Collaboration and enrollment across institutions through consortia and networks also requires focus on enrollment of AYA patients [6]. An approach to enhance enrollment is through the establishment of the NCI's NCTN in 2014 (Figure 1). The NCI developed the NCTN, which is a network of four US‐based adult cooperative CCT groups, one Canadian adult cooperative clinical trials group, and a pediatric cancer cooperative clinical trials group that operates in the United States, Canada, and beyond. The NCTN consists of more than 2200 sites across the United States, Canada, and internationally. NCI cooperative group enrollment of pediatric and AYA patients has improved since the NCTN was formed [7].

The NCTN coordinates and conducts controlled clinical trials from which new standards of care may be established, provides the clinical data for approval of new therapies by the Food and Drug Administration, tests new treatment approaches, and validates new biomarkers. NCI supports multiple centralized services for the NCTN, including the Central Institutional Review Board (CIRB) and the Cancer Trials Support Unit (CTSU). The CIRB is an important component of NCI's clinical trials system that adds speed, efficiency, and uniformity to ethics reviews as well as access for trials for rare cancers. NCI‐funded service that provides clinical investigators and their staff with online access to NCTN trials and allows investigators to efficiently register new patients.

**FIGURE 2:**
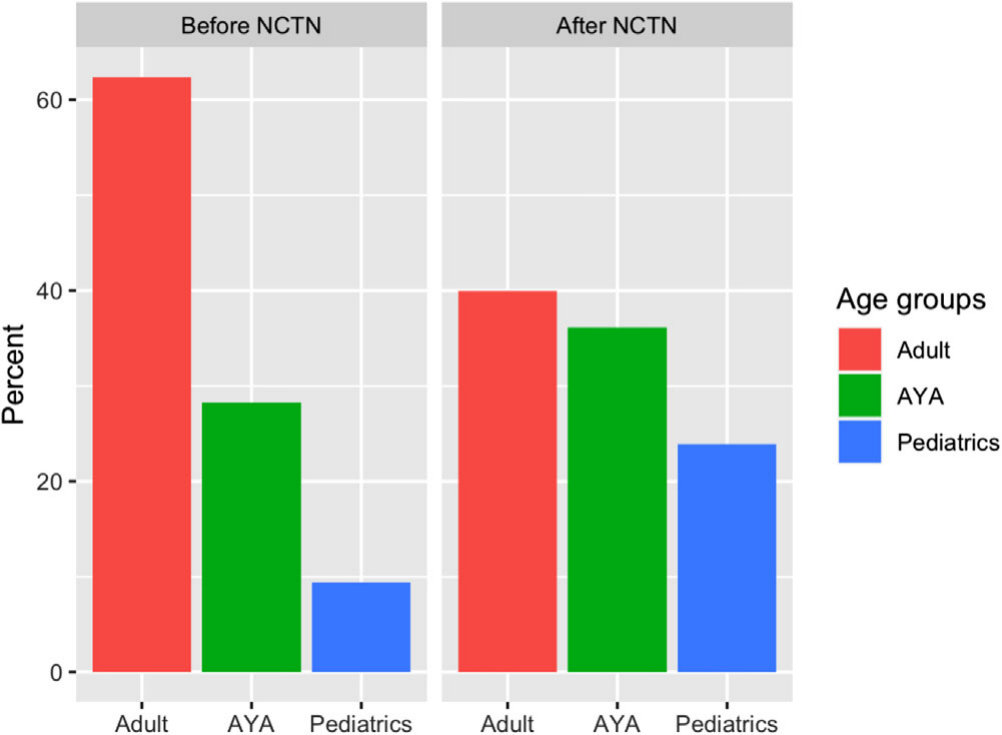
Enrollment onto National Cancer Institute Cooperative Group lymphoma trials before and after formation of the National Clinical Trials Network (NCTN), demonstrating an increase in percentage of adolescent and young adult (AYA) and pediatric patients [7].

The NCI‐supported central IRB has been one of the contributing factors to increased enrollment in the NCTN. This enables institutions to activate an NCTN trial when a patient presents at their institution, reducing time and resources needed to activate trials beforehand. The collaboration within NCTN groups also leads to efficiencies and cost savings. By sharing resources through a collaborative approach, each group can support trials led by other groups to allow member sites to conduct a more robust portfolio of trials. Improvement in enrollment with the creation of the NCTN demonstrates the need for an organized approach to translational and clinical research involving the AYA population. Figure 2 demonstrates increased AYA enrollment in NCI Cooperative Group phase 2/3 and 3 lymphoma trials after the formation of the NCTN. Table 1 describes current cooperative lymphoma trials available through the NCTN. Historically these trials were pediatric or adult specific, but with the advent of the NCTN in 2014, collaborative trials, including S1826 (NCT03907488), have been both possible and successful [7]. S1826 is the largest HL trial in NCTN history and is an important milestone in harmonizing pediatric and adult standard of care [17]. At the time of submission, NCTN has a portfolio of 13 open lymphoma trials applicable to the AYA population. In addition to these national efforts, more international collaboration would further these goals.

**TABLE 1:** National Clinical Trials (NCT) Network lymphoma trials open at time of submission.

Protocol number	Phase	Diagnosis	NCT number
A051901	I	Primary Central Nervous System (CNS) lymphoma	NCT04609046
E4412	I/II	Classical Hodgkin lymphoma	NCT01896999
S2114	II	Diffuse large B‐cell lymphoma	NCT05633615
S2207	II	High‐grade B‐cell lymphoma	NCT05890352
S1608	II	Follicular lymphoma	NCT03269669
A051902	II	Peripheral T‐cell lymphoma	NCT04803201
S1905	II	Precursor T lymphoblastic lymphoma	NCT04315324
A042001	II	Precursor B lymphoblastic lymphoma	NCT05303792
A051701	II/III	Diffuse large B‐cell lymphoma	NCT03984448
ANHL1931	III	Primary mediastinal B‐cell lymphoma	NCT04759586
EA4151	III	Mantle cell lymphoma	NCT04765111
S1925	III	Chronic lymphocytic leukemia/small lymphocytic lymphoma	NCT04269902
AHOD2131	III	Classical Hodgkin lymphoma	NCT05675410

Participation in clinical trials is necessary in order to improve survival in high‐risk patient populations, provide access to novel therapies, and study biology of distinct populations included in each study. Although recruiting AYA patients is likely to take time, this demographic is an important subgroup to understand and increased enrollment in available trials is imperative to ensure that they are treated with optimal therapeutic approaches. Furthermore, long‐term toxicities and appropriate mitigation strategies will never be well understood if there is insufficient enrollment and monitoring of these patients. Facilitators to trial enrollment, based on a COG AYA survey of institutions, were strong communication between pediatric and medical oncology within an institution, supportive research infrastructure and AYA champions [18]. From an institutional perspective, therefore, a structured AYA approach should be taken that includes collaboration between pediatric and adult providers, AYA working groups, and AYA coordinators in order to enhance AYA enrollment.

Addressing and rectifying these identified issues requires communication and collaboration between all stakeholders mentioned, including regulatory agencies, sponsors, academic institutions, IRBs, community providers, patients, and patient advocates. Hence, forward‐thinking solutions with multi‐stakeholder involvement need to be explored to support agreed upon standards of care for treatment and monitoring of disease, definitions of disease response, as well as administrative requirements such as the establishment and maintenance of LTFU registries. The most important way to move AYA research forward is to continue to improve awareness among drug developers (to realize the necessity of AYA studies), physicians (to facilitate enrollment and encourage sponsors to include these patients), regulators (to recommend where AYA populations should be included in studies and to be willing to include younger patients in adult trials), and patients (to educate about clinical studies and motivate participation). Through collaboration of all stakeholders in structured frameworks, outcomes for the AYA population can be improved with increased access to investigational or novel agents through clinical trials.

## AUTHOR CONTRIBUTIONS

Keri Toner wrote the manuscript. Heather Wasserstrom, Carl E. Allen, John Leonard, Shweta Jain, Brad Kahl, Jonathan W. Friedberg, Nita L. Seibel, and Kara Kelly provided initial concept and critically revised the manuscript.

## CONFLICT OF INTEREST STATEMENT

Keri Toner, John Leonard, and Nita L. Seibel have no conflicts of interest to disclose. Shweta Jain is an employee of Seagen Inc. Heather Wasserstrom is an employee at Bristol Myers Squibb. John Leonard provides consulting advice for Abbvie, Astellas, AstraZeneca, Bayer, Beigene, BMS, Calithera, Constellation, Caribou Biosciences, Eisai, Lilly, Epizyme, Genmab, Grail, Incyte, Janssen, MEI Pharma, Merck, Mustang Bio, Novartis, Pfizer, Roche/Genentech, Seagen, Second Genome, and Sutro. Carl E. Allen is on advisory boards for SOBI and OPNA and has research support from Genentech. Kara Kelly is on scientific steering committee of Merck and Seagen.

## FUNDING INFORMATION

Lymphoma Research Foundation.

## ETHICS STATEMENT

Ethics approval was not obtained due to the review nature of this paper without original research generated.

## PATIENT CONSENT STATEMENT

This manuscript did not include original patient research.

## PERMISSION TO REPRODUCE MATERIAL FROM OTHER SOURCES

Figures from NCI with permission.

## CLINICAL TRIAL REGISTRATION (INCLUDING TRIAL NUMBER)

The authors have confirmed clinical trial registration is not needed for this submission.

## Data Availability

All data that support the findings of this study are included within this paper.
